# L2M1 and L2M2 Acquisition of Sign Lexicon: The Impact of Multimodality on the Sign Second Language Acquisition

**DOI:** 10.3389/fpsyg.2022.896254

**Published:** 2022-06-10

**Authors:** Krister Schönström, Ingela Holmström

**Affiliations:** Department of Linguistics, Stockholm University, Stockholm, Sweden

**Keywords:** sign language (SL), second language (L2) acquisition, multimodality, lexicon, cross-linguistic influence (CLI), transfer

## Abstract

In second language research, the concept of cross-linguistic influence or transfer has frequently been used to describe the interaction between the first language (L1) and second language (L2) in the L2 acquisition process. However, less is known about the L2 acquisition of a sign language in general and specifically the differences in the acquisition process of L2M2 learners (learners learning a sign language for the first time) and L2M1 learners (signers learning another sign language) from a multimodal perspective. Our study explores the influence of modality knowledge on learning Swedish Sign Language through a descriptive analysis of the sign lexicon in narratives produced by L2M1 and L2M2 learners, respectively. A descriptive mixed-methods framework was used to analyze narratives of adult L2M1 (*n* = 9) and L2M2 learners (*n* = 15), with a focus on sign lexicon, i.e., use and distribution of the sign types such as lexical signs, depicting signs (classifier predicates), fingerspelling, pointing, and gestures. The number and distribution of the signs are later compared between the groups. In addition, a comparison with a control group consisting of L1 signers (*n* = 9) is provided. The results suggest that L2M2 learners exhibit cross-modal cross-linguistic transfer from Swedish (through higher usage of lexical signs and fingerspelling). L2M1 learners exhibits same-modal cross-linguistic transfer from L1 sign languages (through higher usage of depicting signs and use of signs from L1 sign language and international signs). The study suggests that it is harder for L2M2 learners to acquire the modality-specific lexicon, despite possible underlying gestural knowledge. Furthermore, the study suggests that L2M1 learners’ access to modality-specific knowledge, overlapping access to gestural knowledge and iconicity, facilitates faster L2 lexical acquisition, which is discussed from the perspective of linguistic relativity (including modality) and its role in sign L2 acquisition.

## Introduction

Our current knowledge of sign second language acquisition is mainly informed by research involving second language (L2) learning of a sign language in a second modality (M2), i.e., it is based mainly on research on hearing adult learners with a spoken language as a first language (L1) who are learning a sign language as an L2 using a different modality. However, knowledge about the same modality (M1) learning of a sign language, i.e., deaf adult learners with an L1 sign language learning a new L2 sign language, is scarce, particularly regarding the acquisition of linguistic structures. Therefore, this study attempts to fill this knowledge gap by looking at sign acquisition in deaf L1 signers learning a new sign language as an L2, namely, Swedish Sign Language (*Svenskt teckenspråk*, STS), and comparing it with the L2 learning of hearing L2 learners of STS. We focused on both L2M1 and L2M2 signers, which are referred to as M1 and M2 signers for ease of reading, as the overall framing of this study is second language acquisition.

One important area of second language acquisition (SLA) is the concept of cross-linguistic influence (CLI) or transfer. This research has shown us that language learners seem to transfer previous language knowledge to another language. The concept and the characteristics of this transfer between the languages among multilingual learners have been widely discussed in the literature (Jarvis and Pavlenko, 2008). For sign languages, earlier research has pointed out the influence of gestural knowledge and iconicity on acquiring a sign language among M2 learners (e.g., Taub et al., 2008; Ortega, 2017; Ortega et al., 2019). However, less is known about the M1 acquisition of a sign language in general and specifically the differences in the acquisition process of M1 learners and M2 learners. This article seeks to contribute to this field.

We aimed to examine how prior modality knowledge influences learning STS as an L2 through a descriptive study of the sign lexicon in retold narratives produced by M1 and M2 learners. With CLI as a framework, we aimed to describe the degree and types of transfer on L2 STS as the recipient language depending on the learners’ source languages (L1/Ln), to understand the effect of multimodality and its role for L2 acquisition. The “Introduction” section of this article describes the sign language lexicon from the perspective of STS. The next section, “Sign Second Language Acquisition (SSLA),” summarizes the core body of research relevant to the scope of this study, including the concept of CLI and previous research on Sign L2 acquisition, which is followed by the “Materials and Methods” section. Finally, results and discussion are presented.

## Sign Language Lexicon

While many sign languages share similar properties, sign linguistics literature contains different theoretical descriptions and classifications pertaining to a variety of sign categories. Thus, the categories have been labeled differently in the sign language literature, depending on their form, meaning, and degree of lexicalization and conventionalism. In our description, we departed from the study’s language, STS. We also attempted to adopt a non-theory-bounded and descriptive approach to describe the sign categories. A sign can be seen to be equivalent to the traditional concept and definition of a word, although there are alternative views on this (e.g., Lepic, 2019). Some researchers suggest that the signed modality (in comparison with the spoken modality) allows for some modality-dependent characteristics, affecting the linguistic structure (e.g., Meier (2002)). First, the nature of signed modality with using manual components (i.e., the hands) and the non-manual components (i.e., facial expressions and body movement) allows for a higher degree of simultaneity in production and for the visual perception of the information. Second, there is the possibility of using the space in the front of the signer to create meaning and reference, and it affects, for example, the lexicon. From a phonological view, Brentari and Padden (2001) described a model of sign language lexicon as three components based on the forms of the signs, i.e., divided into *non-native* (or foreign) and *native* signs. The native signs category is, in turn, divided into a *core lexicon* and a *spatial lexicon*. The non-native lexicon is formed based on the manual alphabet, e.g., fingerspelling. The core lexicon includes signs that are lexical and conventionalized and typically included in a sign language dictionary, e.g., lexical signs. The spatial lexicon includes signs that are partly conventionalized in form. The use of spatial lexical items requires context to be fully understood, e.g., depicting signs [also labeled as, e.g., classifier predicates, polycomponential signs, and depicting constructions in the literature (e.g., Liddell, 2003; Schembri, 2003; Cormier et al., 2012 for comprehensive overviews)]. On a somewhat similar matter, Johnston and Schembri (2010) (also refer to Johnston and Ferrara, 2012; Hodge and Johnston, 2014) divided the sign lexicon into three components based on their degree of lexicalization: *lexical*, *partly lexical*, and *non-lexical*. Lexical signs correspond to the signs that can be said to be most conventionalized in form and meaning and equivalent to the notion of the “word” in spoken languages and that are listed in dictionaries, e.g., the STS dictionary. Partly lexical signs are signs that are partly conventionalized in form and meaning. Those signs often require context to be understood. Lexical signs include depicting signs and pointing. Finally, non-lexical signs are signs that are at least conventionalized in form and meaning. In this study, gestures and other manual or non-manual acts are included, e.g., they vary widely in form and meaning and are highly dependent on contextualization.

We will now define the sign categories that we have considered in this study: lexical signs, fingerspelling, pointing, depicting signs, and gestures.

*Lexical signs* are the signs that can be found in the STS dictionary, i.e., “frozen signs” that are conventionalized in form and meaning. STS dictionary work has been conducted since the 1990s at Stockholm University and consists today of approximately 20,000 recorded and officially published signs. The lexical signs in the dictionary have been collected through observations of signs used in the deaf community (through, i.e., social media), the media (TV broadcasts in sign language), and signs found in the Swedish Sign Language Corpus. This large STS dictionary has provided us with a source to consult in order to ensure that the signs we identified in this study can be considered lexical signs. Often these signs are accompanied by a specific mouth action that can be either sign language-based (i.e., mouth gestures) or borrowed from Swedish (mouthing) (e.g., Crasborn et al., 2008; Mesch et al., 2021 for closer descriptions of mouth actions in sign languages and STS). In STS, mouthing is a frequent mouth action category, especially functional, and is used to distinguish between ambiguous lexical signs, i.e., manual sign homonyms.

*Fingerspelling* is an alternative usage to lexical signs and comes in two different formats. First, *full fingerspelling* is especially used to express names and concepts and to borrow words from spoken languages (not only Swedish). Full fingerspelling does not follow the standard phonological configuration of a sign (e.g., in the parameters of handshape, movement, location, and orientation). Then, there is *lexicalized fingerspelling*, where the sign has its origin in fingerspelling but has been reduced and conformed closer to the standard phonological formation of a lexical sign (cf. Battison, 1978, on fingerspelled words).

*Depicting signs* include signs not listed in the STS dictionary and have been called multimorphemic constructions (e.g., Wallin, 1996, in terms of “polysynthetic signs”), whose form and meaning depend on contextualization. Handshape types are a key component consistently reported in the literature on depicting signs and are used to describe such signs. As in earlier accounts using this type of data (e.g., Schönström and Mesch, 2019), we departed from three main categories of handshape units: entity, handle, and descriptor in order to be able to identify the depicting signs in our data, and the representation of the movement that can be linked to the main categories of movement or existing. This category may fall within the frame of morphosyntax, but in our presentation we have treated this category as part of STS lexicon, departing from a broader application of the use of signs.

*Pointing* is another category of sign that is a recurring component of many sign languages and is primarily linked to the use of the INDEX hand. Its physical form is simply pointing toward different locations or at different objects. But in sign languages, pointing is more refined and part of the sign lexicon, often functioning as pronouns (e.g., Cormier et al., 2013). Its meaning depends on the location in the signing space to where the sign points. It can be considered a partly lexical sign.

As *gestures*, we have counted such acts that vary widely in form and meaning and are not part of any categories described above, such as palm-up gestures and “come here” gestures.

Frequency is essential when studying sign categories in narratives (e.g., Johnston, 2012). By analyzing how often different sign categories appear in the study data, we can learn how common one category is in comparison with other categories. Analyzing frequency also allows us to see if there are differences between different signers, such as L1, M1, and M2 signers. For example, L1 data can inform us about how common it is to use lexical signs and depicting signs in narratives and thus tell us how M1 and M2 signers relate to these data.

## Sign Second Language Acquisition (SSLA)

Although research on SSLA is a growing field of interest among scholars, most of the research to date has focused on M2 adult learners, i.e., primarily hearing adults with an L1 spoken language that is learning a sign language as an L2 (e.g., Schönström, 2021). There are few studies of M1 learners, i.e., primarily deaf adults with an L1 sign language learning another sign language as an L2. We have not found any study on the acquisition of sign lexicon among M1 signers except for a study by Koulidobrova (2019), who studied argument omission in M1 signers.

It is essential to consider the modality effect for the two groups of learners; M2 learners have to learn to express language in a totally different way than previously, while M1 learners already have the skills to express themselves through the visual-gestural modality. With this in mind, it is necessary to understand that the SSLA process can be partly different, depending on whether the learners are M1 or M2 learners.

Not only do M2 learners have to learn the sign language itself but they must also learn how to express the language. According to Woll (2013), learners may experience difficulties adapting the visual-gestural modality, i.e., using the body, facial expressions, gaze, etc., to produce language. Other features may also impact their learning. For example, they already have motoric skills in their fingers, hands, and arms, but they must learn how to use them to express signs with correct phonology. Another new learning feature is non-manual grammar expressed through, e.g., moving eyebrows. The learners already use these movements in their daily life. However, when learning a sign language, they need to understand the movements as grammatical markers and learn to use them correctly. The same applies to the use of gestures. Speakers of a spoken language often use gestures in different ways. Sometimes, these gestures appear together with speech, co-speech gestures, and sometimes, they are used to complement or replace speech (Özyürek, 2012). Several studies have suggested that such gestural knowledge used in spoken languages can be beneficial for M2 learners’ acquisition of sign languages (Casey and Emmorey, 2009; Chen-Pichler and Koulidobrova, 2015; Ortega et al., 2019; Marshall et al., 2021).

An apparent feature in sign languages is that a considerable number of signs are highly iconic, meaning that the formations of signs correspond to or are transparent representations of the form, shape, or action of reality, i.e., a ball’s form or the action of carrying a bag. Ortega and Morgan (2015a,b) found iconicity to have both advantages and disadvantages for M2 learners of British Sign Language (BSL). The advantages were that it was easier for the learners to understand and memorize the signs by connecting them to reality (cf. Baus et al., 2012). Simultaneously, the iconicity led the learners to fail to note how the sign is performed correctly phonologically, presumably because they find the signs “easy.” Thus, the iconicity may cause disadvantages. When comparing a range of different studies on the iconicity impact on sign L2 learning, Ortega (2017) confirmed that iconicity has both positive and negative impacts on learning. His compilation shows that iconicity seems to positively affect the sign’s conceptual-semantic feature but not its linguistic structure.

A cross-sectional study by Schönström and Mesch (2019) investigated the development of depicting sign use of M2 signers with deaf L1 signers as a benchmark. Their results revealed that M2 signers tend to stick to lexical signs rather than depicting signs compared with L1 signers, but that the proportion of depicting signs grows with acquired sign proficiency and experience. In addition, the M2 signers exhibit the use of depicting signs early on, which confirms previous results on M2 learning of depicting signs as reported by Marshall and Morgan (2015) for BSL and by Boers-Visker (2020) for Dutch Sign Language (NGT).

The spatial structure of sign languages is another characteristic that the research body has identified as difficult to acquire for M2 learners (e.g., McKee and McKee, 1992; Ferrara and Nilsson, 2017; Shield and Meier, 2018; Boers-Visker, 2020; Gulamani et al., 2020). The space in front of the body can be used in a range of ways, not only for articulating lexical signs but also for grammatical and discourse purposes (Perniss, 2012; Boers-Visker, 2020). Such use of the space is initially unfamiliar for new signers who initially do not know that there is a signing space in front of their body. Ferrara and Nilsson (2017) found in their study that M2 learners of Norwegian Sign Language struggled to use the signing space and instead relied on lexical signs, i.e., the learners chose a familiar, sequential strategy with one lexical sign after another, rather than the unfamiliar spatial strategy that places the sign in a specific location or direction.

As M2 signers are often initially unfamiliar with the use of face and body to express language, sign language instruction often includes modality-specific training (Holmström, 2019, 2021, also refer to McKee and McKee, 1992). Holmström (2019) found that teaching a university STS beginners’ course largely consisted of modality-specific metalinguistic information. For example, the teachers told the students about the differences between spoken and sign languages in expression, perception, and grammar. In addition, the teacher made them aware that the view of signs differs for the signer and the addressee. In a follow-up study, Holmström (2021) further examined STS teaching and found that during the initial stage of their learning, students were particularly trained to make and keep eye contact, get attention, and use visual turn-taking. She also found that a large amount of the teaching consisted of exercises in learning and using iconicity, spatiality, and simultaneity in STS. The students in this study said that they initially found the exercises very strange, but gradually, they made them more comfortable expressing language with face and body. This indicates that M2 learners benefit from modality-specific training to develop their L2 and move away from the linear and lexical production into a more spatial one, with depicting signs and constructed action.

### Transfer/Cross-Linguistic Influence

In comparison with SSLA, there is a vast body of SLA research on spoken language from a wide range of perspectives. An important topic of SLA research has been the study of transfer or cross-linguistic influence (CLI) and its role in L2 acquisition, and there is an extensive body of research on CLI in the SLA literature (for reviews, refer to Odlin, 1989; Jarvis and Pavlenko, 2008). CLI can be defined as the influence of an L2 learner’s prior knowledge of one or more languages on the processing and use of the new language. Typically, CLI research seeks to answer the question of how prior knowledge of one or more languages shapes learning a new language. *Transfer* is one common concept of outcome in CLI. According to Odlin (1989), a transfer is seen as a result of the influence based on the similarities and differences between the target language (i.e., the L2) and the other previously acquired language. Typically, as regards directionality, it has been studied under the framing of the influence of L1 on L2 learning (i.e., *forward transfer*). However, more recent studies have also included perspectives on the influence of L2 knowledge on learning additional languages (L3, L4, etc.) (i.e., *lateral transfer*). Furthermore, studies have suggested that learners can transfer knowledge from an L2 to L1 (i.e., *reverse transfer*) (Jarvis and Pavlenko, 2008).

One of the main factors influencing the degree of CLI is the learner’s perceived cross-linguistic similarity between the languages (i.e., the L1 and the L2). CLI is more likely to happen when the learner perceives a similarity between the L1 and the L2 rather than when the learner perceives the languages as different. Ringbom (2007) suggested that cross-linguistic similarity facilitates learning of the new language as it gives the learners the ability to link words and/or structures to other similar words or structures. Furthermore, Ringbom claimed that linguistic and typological distance between the languages (i.e., the L1 and the L2), i.e., linguistic relativity, plays an essential role in the CLI processes. Researchers have found and discussed different types of transfer. The earliest accounts of transfer focused on errors in the target language caused by transfer, i.e., *interference* or negative transfer. However, later research has pointed out that the ultimate outcomes of CLI are often positive. Moreover, learners’ perceived assumptions about the similarities and/or differences between source and recipient languages can lead to underproduction or overproduction of structures in the recipient language.

Previous CLI research has shown that transfer can occur in several linguistic areas (e.g., phonology, vocabulary, and syntax). Moreover, it can also be manifested in more cognitive matters, i.e., through the learners’ knowledge of how different meanings or concepts are expressed (e.g., time and location). Furthermore, Jarvis and Pavlenko suggested a ten-dimensional model of CLI types based on its characteristics, i.e., (a) areas of language knowledge, (b) directionality, (c) cognitive level, (d) type of knowledge, (e) intentionality, (f) mode, (g) channel, (h) form, (i) manifestation, and (j) outcome (Jarvis and Pavlenko, 2008, p. 20ff).

Even within SSLA research, CLI has been a subject of interest for several researchers. Some researchers have pointed out limited possibilities of a “physical” transfer between a spoken and sign language, at least with regard to phonology (Rosen, 2004; Bochner et al., 2011; Ortega and Morgan, 2015a,b). However, Chen Pichler (2010) suggested a more abstract treatment of the notion of phonology, allowing for an analysis of the previous gesture skills in M2 learners and its influence on L2 ASL phonology. Chen Pichler found instances of unmarked handshapes in L2 ASL, where marked handshapes were target forms, and suggested that erroneous use of unmarked handshapes was a result of transfer from M2 learners’ gestural knowledge, affecting ASL phonology. Furthermore, as described above, Ortega and Morgan (2015a,b) and Ortega et al. (2019) suggested that there are effects of transfer on BSL phonology originated in the learners’ prior knowledge of gesture and concepts linked to iconicity. As a result, this prior knowledge leads to positive and negative effects on BSL phonology. Ortega et al. (2019) suggested this to be explained in terms of manual cognates, i.e., there is a perceived similarity between the gestures and signs, which is scaffolding the learners’ learning of the sign lexicon. Furthermore, the development of spatio-visual skills in M2 ASL learners has been studied by Taub et al. (2008), who examined the use of classifier structures (i.e., depicting signs) (in third-person discourse structures), constructed action (in first-person discourse structures), and location in signing space. As the use of gestures has been shown to have an important role in spoken languages and its use of the spatial domain, Taub et al. (2008) suggested that there is a possibility that some previous spatio-visual knowledge in the source language, for example, the knowledge of using direct speech/constructed dialog, could be transferred to L2 ASL. However, they found no such transfer patterns regarding first-person discourse (i.e., the use of constructed action), but some transfer patterns regarding third-person discourse (i.e., the use of classifier structures) and on the use of spatial location structures. Taub et al. (2008) suggested that ASL learners focus on vocabulary items (which inhibit the use of constructed actions) and transfer the use of iconic co-speech gestures into classifier-like structures and that preexisting skills in using location in gesture are transferred to the use of location in the signing space in L2 ASL. In a corpus-based study on the use of mouth actions in M2 learners, Mesch and Schönström (2021) compared the use of mouth actions in M2 learners and L1 signers of STS. They found an overproduction of the mouthing category of mouth actions (i.e., borrowed-in mouthing of Swedish) in M2 learners, suggesting that it was an effect of transfer from L1 Swedish into L2 STS.

When combined, our current but limited knowledge about CLI in SSLA has been limited to M2 learners. To broaden our understanding of CLI in SSLA, a study involving both M1 and M2 learners would be fruitful. Our working hypothesis is that M1 learners encounter the learning of iconic and spatio-visual skills positively compared with M2 learners due to cross-linguistic and modality similarity. M2 learners encounter a challenge when learning such structures, and furthermore, M2 learners approach the learning of non-spatial lexicon (e.g., lexical signs and fingerspelling) differently to M1 learners due to the perceived structural similarity to words of L1 Swedish. This will have different outcomes in the produced sign lexicon between the learner groups due to different CLI sources in their processing and use of L2 STS.

## Materials and Methods

Using a descriptive mixed-methods framework, 24 narratives from adult M1 (*n* = 9) and M2 (*n* = 15) learners were analyzed. This study uses data from two research projects focusing on adult L2 learners. The first one is the ongoing project Mulder (the multilingual situation of deaf refugees in Sweden) with a focus on deaf M1 learners. For project Mulder, data were collected at four folk high schools (independent adult education colleges) with programs for deaf migrants learning STS and Swedish. The second is the previous project TATE (Från tal till tecken–att lära sig Svenskt teckenspråk som andraspråk [From speech to sign–learning Swedish Sign Language as a second language]), focusing on hearing M2 learners. Within the project TATE, an STS as L2 corpus was constructed comprising data from M2 learners (Schönström and Mesch, 2017).

In both projects, the participants performed an elicitation task consisting of a short clip from the movie “The Plank.” Two men struggle to carry a plank through an urban area in the minute-long clip. The main event involves the plank going through a window into a bar, causing a glass of beer to fall out into a bucket outside the bar. A misunderstanding then arises between the window cleaner and the person whose beer it is. This clip was chosen to elicit linguistic constructions related to depicting signs and constructed actions, as well as spatial constructions. In project TATE, deaf L1 signers were recruited to perform the same task and create a control group to compare with the M2 signers. The data from Mulder and the STS as L2 corpus allow us in this study to compare narratives from M1 and M2 signers, as well as L1 signers.

The narratives were transcribed using ELAN and coded by sign type. A transcription protocol developed by Wallin and Mesch (2018) was used in the annotation work. Also, a further developed protocol for L2 analysis (Mesch and Schönström, 2018) was used. This included transcription through a controlled vocabulary list, including information about sign types associated with every sign in the list (lexical sign, pointing, depicting sign, fingerspelling). The manual signing was transcribed concerning using the dominant hand and non-dominant hand representing the sign glosses. Transcription of the sign language data is, in general, time-consuming. However, thanks to the available STS L2 corpus comprising M2 data, we were able to compare our new M1 data obtained within the Mulder project with the M2 data from the corpus. Several people have contributed to the manual transcription work of the sign language data. All coders have been deaf native STS users and students in sign linguistics or senior sign linguistic researchers. For the STS as L2 corpus data (M2 data), deaf research assistants were hired to code the sign glosses with the project team (of which the first author was part). The transcription was later controlled by a deaf senior researcher of the team. For the Mulder project (M1 data), the same procedure was applied. A deaf sign linguistic student coded the signs together with the first author of this study. However, no inter-rater reliability data are available. Instead, the work with the coding is integrated into a teamwork style with discussions within the team. The first author also controlled all the coding in order to ensure consistency. In the next step, the frequencies and distribution of the signs were categorized by gloss and sign types (Table 1).

**TABLE 1:** Coding of sign type categories.

Sign type	Sub-category	Example
Lexical signs	STS signs	POLICE “police”
	International/Ln signs	DRINK@it “drink”
Depicting signs	Entity	ENTITY[handshape]+MOVE “movement of an entity”
	Handle	GRIP[handshape] +HANDLE “handle with grip (of an entity)”
	Descriptor	SIZE[handshape] +SPECIFY “size and shape specifying”
Pointing		INDEX “pointing to self”
Fingerspelling		ÖL@b “beer”
Gesture		HAND-WAVE@g “wave with hands”

Furthermore, we also created a row for the qualitative analysis of instances of CLI for the M1 data, i.e., the negative transfers. Our analysis was limited to the use of signs. The analysis of the negative transfers was explorative, i.e., the authors of the study, both deaf and fluent in STS, analyzed the narratives of the signers and identified instances of what we interpreted as negative transfer and coded them as (1) mouth transfer, (2) lexical transfer, and (3) handshape transfer (refer to the “Instances of cross-linguistic influence (CLI) in M1 and M2 signers” section for further description of the analysis and result).

### Participants

Project Mulder recruited data from a considerable number of M1 signers, but to make the comparisons as equal as possible with the M2 learners we restricted our group to nine M1 signers, i.e., five male signers and four female signers, *M*_*age*_ = 36.7 years, *SD* = 4.6, range 30–45 years. All nine participants were born into deaf families and have acquired a sign language from birth. They also have a fundamental educational background, i.e., they have undergone at least elementary school, and most of them have also undergone some kind of secondary school level. Table 2 shows the participants’ background data. We also conducted a non-verbal cognitive test, Kaufmann Brief Intelligence Test, Second Edition (KBIT-2; Kaufman and Kaufman, 2004), which revealed that the nine participants’ IQ profiles are average. All participants were recruited through the four folk high schools, and most of them had been enrolled at the schools for around 3–7 months.

**TABLE 2:** M1 participants in the study.

ID	Gender	Age	L1	Years of schooling	Length in Sweden at data collection
203	Female	40	Iranian SL	12	6 months
205	Male	30	Italian SL	15	6 months
206	Male	31	Lithuanian SL	11	6 months
207	Female	45	Latvian SL	12	5 years
210	Female	37	Polish SL	15	3 months
211	Male	36	Polish SL	12	3 months
212	Male	39	Polish SL	12	3 months
213	Male	38	Polish SL	14	3 months
306	Female	34	Russian SL	10	6–7 months

Data from the M2 participants were obtained from the STS as L2 corpus. All participants are hearing adults with Swedish as L1 and attend a sign language interpreting program at the university level. None of the participants had learned a sign language before enrollment. As the STS as the L2 corpus is longitudinal, we decided to depart from data from a group that has studied sign language for approximately 5–6 months as the M1 participants had learned STS for approximately 3–7 months (with one exception of one who has been in Sweden for 5 years). The group of M2 learners consists of 15 students: 2 male students and 13 female students, *M*_*age*_ = 23.9 years, *SD* = 5.1, range 19–40 years.

As a control group, data from L1 signers, one male student and eight female students, *M*_*age*_ = 27.6 years, *SD* = 11.22, range 20–50 years, were obtained from the STS as L2 corpus.

## Results

The results are presented below. First, we accounted for the frequency and distribution of signs used in the groups. Second, we accounted for instances of cross-linguistic influence found in the data.

### Frequency and Distribution of Signs

The frequency and distribution of sign categories by group are presented below. Table 3 shows the group’s frequency of signs, including mean, standard deviation, standard error, range (min–max), and 95% confidence intervals. N signs refer to the total number of glosses transcribed in the analysis. It also demonstrates the mean length of the narratives by group. This includes all the glosses, including held signs and unclear signs used in the narratives. In addition, the M1 group produced a type of sign that we have coded as foreign lexical signs, i.e., signs from other sign languages, such as their L1 or international sign. In total, we found 22 instances of foreign signs for the whole group of M1 signers, *M* = 2.4, *SD* = 1.7, range 0–4. No such use was observed in the L1 and M2 groups.

**TABLE 3:** Mean frequency and distribution of signs and sign categories [lexical signs, depicting signs (DS), fingerspelling, pointing, and gesture] in group level.

		*N*	*M*	SD	S.E.	95% CI for M		Min	Max	Proportion of total signs
						Lower	Upper			
N Signs	M1	9	99.3	48.4	16.1	62.2	136.5	31	187	
	M2	15	116.7	43.8	11.3	92.4	140.9	60	183	
	L1	9	156.4	40.9	13.6	125.0	187.9	81	208	
Lexical Signs	M1	9	35.9	20.3	6.8	20.3	51.5	11	77	43.8%
	M2	15	65.8	22.4	5.8	53.4	78.2	30	111	63.4%
	L1	9	77.4	25.1	8.4	58.2	96.7	39	114	54.8%
Depicting Signs	M1	9	34.3	16.2	5.4	21.9	46.8	14	60	41.9%
	M2	15	18.5	12.4	3.2	11.7	25.4	1	41	17.9%
	L1	9	38.7	11.5	3.8	29.8	47.5	21	55	27.3%
Fingerspelling	M1	9	2.1	1.7	0.6	0.8	3.4	1	6	2.6%
	M2	15	10.1	5.4	1.4	7.1	13.1	5	21	9.8%
	L1	9	10.8	4.5	1.5	7.3	14.3	6	20	7.6%
Pointing	M1	9	7.4	6.3	2.1	2.6	12.3	0	17	9.1%
	M2	15	6.2	6.4	1.7	2.6	9.8	1	23	6.0%
	L1	9	12.2	6.2	2.1	7.4	17.0	3	23	8.6%
Gesture	M1	9	2.1	2.1	0.7	0.5	3.8	0	7	2.6%
	M2	15	3.1	2.4	0.6	1.8	4.4	0	7	3.0%
	L1	9	2.3	2.0	0.7	0.8	3.9	0	5	1.6%

The last column in Table 3 presents the distribution of the sign categories lexical signs, depicting signs (DS), fingerspelling, pointing, and gestures, in mean and percent of the total number of signs (i.e., the categories combined) by the groups M1, M2, and L1 signers. The distribution of the sign categories differs between the groups. Regarding the category of *lexical signs*, M2 signers exhibit the highest proportion (63.4%), followed by L1 signers (54.8%) and M1 signers (43.8%). M1 signers exhibit the highest proportion for the category *depicting signs*, with 41.9%, followed by L1 signers (27.3%) and M2 signers (17.9%). M2 signers have the highest proportion regarding *fingerspelling* with 9.8% followed by L1 signers and M1 signers with 7.6 and 2.6%, respectively. *Pointing* was mostly used by L1 and M1 signers with 8.6 and 9.1%, respectively, compared with M2 signers (6.0%). Finally, *gestures* were generally minimal for all the groups with 2.6, 3.0, and 1.6% for M1, M2, and L1 signers, respectively.

To determine if there are any statistically significant differences between the groups in category means, a one-way ANOVA was run. A Shapiro-Wilk’s test for normality revealed that means for lexical signs and depicting signs were normally distributed, but that means for fingerspelling (violated for M1 and M2 group), pointing (violated for M2 group), and gesture (violated for M1 group) were not normally distributed. Levene’s test for equality of variances confirmed the assumptions of homogeneity of variances for the following categories: lexical signs (*p* = 0.0598), depicting signs (*p* = 0.281), pointing (*p* = 0.967), gesture (*p* = 0.0570), but was violated for fingerspelling (*p* = 0.034). Welch’s ANOVA revealed that there was a statistically significant difference between the groups in lexical signs *F*(2,17.301) = 8.612, *p* = 0.003, depicting signs *F*(2,16.776) = 8.555, *p* = 0.003, and fingerspelling *F*(2,16.558) = 24.333, *p* < 0.001, but not for pointing *F*(2,17.561) = 2.569, *p* = 0.105 and gesture *F*(2,18.071) = 0.672, *p* = 0.523.

To explore the contrasts between the groups for each category, Bonferroni *post hoc* analysis was carried out on lexical signs and depicting signs. For lexical signs, the difference between M1 and M2 [−29.91, 95% CI (−54.09 to −5.73)], and M1 and L1 [−41.56, 95% CI (−68.59 to −14.52)] was statistically significant with *p* = 0.011 and *p* = 0.002, respectively, but not between L1 and M2 [11.64, 95% CI (−12.53 to 35.82), *p* = 0.695]. For depicting signs, the difference between M1 and M2 [15.80, 95% CI (1.55 to 30.05)], and L1 and M2 [20.13, 95% CI (5.88 to 34.38)] was statistically significant with *p* = 0.026 and *p* = 0.004, respectively, but not between L1 and M1 [4.33, 95 % CI (−11.60 to 20.27), *p* = 1.00]. For the category fingerspelling, Games-Howell *post hoc* analysis revealed that the difference between M1 and M2 [−8.02, 95% CI (−12.80 to −3.25)] and M1 and L1 [−8.67, 95% CI (−13.06 to −4.27)] was statistically significant with *p* < 0.001 and *p* < 0.001, respectively, but not between L1 and M2 [0.64, 95% CI (−4.57 to 5.86), *p* = 0.947].

In terms of over- and underproduction of target STS forms, the results suggest a modality effect on L2 acquisition based on the proportions of specifically sign language-specific patterns such as depicting signs and signs more closely related to spoken languages such as lexical signs and fingerspelling. It also indicates that lexical signs are under-produced among the M1 group, which we interpreted as they still are struggling with the learning of lexical signs.

Our analysis of the M1 narratives also revealed some interesting qualitative patterns that are suggested as a link to cross-linguistic influence, which will be elaborated further in the next section.

### Instances of Cross-Linguistic Influence (CLI) in M1 and M2 Signers

In our qualitative analysis of M1 data, we found interesting patterns of within-modality cross-linguistic influence. As previously mentioned, we focused on negative transfers, i.e., non-target forms of STS that we have identified as transfers from other sign languages.

**FIGURE 1:**
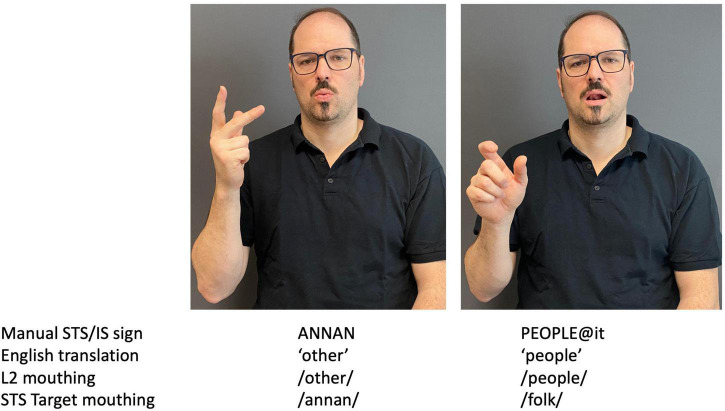
Examples of mouth transfer.

Regarding the M1 learners, we identified 56 instances of negative transfers, where we then conducted a further qualitative analysis. In this study, we identified three types of transfer, namely, mouth transfer, lexical transfer, and handshape transfer. Mouth transfer is when the participants use mouth actions from other language(s) that they know (either an L1 or an Ln). For example, we observed that the learners could add mouthing from English or mouthing from their national spoken languages while producing STS. This seems to happen mostly when the target STS sign’s mouthing is based on Swedish. It was not linked to the manual signing; it could either be a lexical STS sign (as in the sign ANNAN) or a lexical Ln sign (an international sign as in PEOPLE@it) (refer to the examples in Figure 1).

**FIGURE 2:**
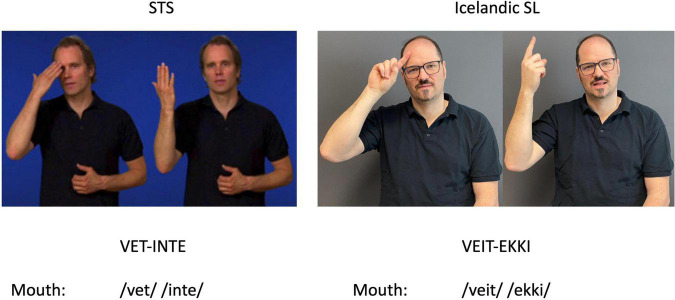
Cross-linguistic similarities of the signs of *vet ekki* and *vet inte* [do not know]. STS image of VET-INTE from STS dictionary ID: 17937 (published with permission).

Interestingly, mouthing transfer happens primarily in combination with STS signs, i.e., the learners are signing STS but use non-target mouthing influenced by their L1 or Ln. Mouthing transfers from English were most common in this type of transfer, but we also identified mouthing of the word *okno* [window] from Polish in participants 211 and 213 and the Icelandic *veit ekki* [do not know] in participant 213. The latter is particularly interesting as this participant first moved from Poland to Iceland and lived there for a few years before moving to Sweden. Thus, this mouth transfer does not come from 213’s L1 but her L2. We also found that the mouthing comes together with the manual Icelandic sign VEIT EKKI [do not know]. The sign (including the mouthing) of *veit ekki* shares some similarities with the equivalent STS sign of *vet inte* [do not know] (Figure 2). There are similarities in the visual surface properties of the mouthing and in the phonological structure of the signs with respect to location and, to some degree, movement, even if the handshape is different.

**FIGURE 3:**
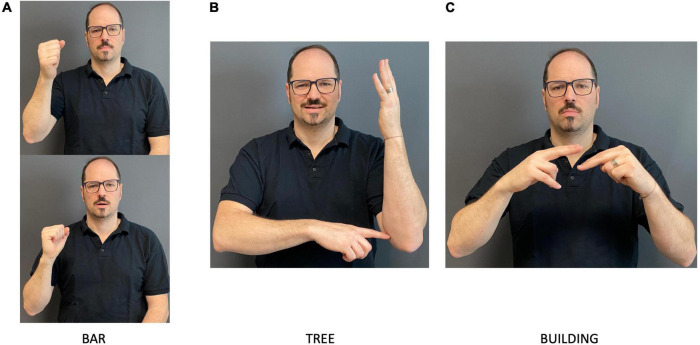
Examples of lexical transfers. The signs are **(A)** BAR, **(B)** TREE and **(C)** BUILDING.

We also observed lexical transfers, i.e., that manual signs from the learners’ L2/Ln language were transferred to STS. The lexical transfers found in the data vary, but some can be identified as signs typically used in international sign contexts, such as the sign for BAR (Figure 3A). Other seem to be variants of signs possibly borrowed from other sign languages, for example, TREE from Lithuanian SL (Figure 3B) and BUILDING probably from ASL (Figure 3C). Due to some of the signs’ depictive characteristics, highly iconic properties, and potential cross-linguistic similarities of unknown SLs, it is not always straightforward to firmly decide the origin of the signs beyond the fact that they are non-STS signs.

Finally, we also observed what we suggest is handshape transfers. Handshape transfers refer to the use of non-STS handshapes or non-target handshapes when producing a lexical or depicting sign. Handshape transfers were the least common and appeared only in two of the learners in our M1 group. For example, we observed the use of the 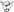 handshape and the 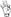 handshape referring to “drinking.” Participant 306, in turn, used a handshape transfer, 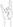, from Russian Sign Language, referring to “window.” These handshapes are not used in such contexts in STS, except for 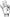 for contexts where drinking from a small teacup.

There was individual variation in the CLI patterns as the participants’ frequency and use of different types of transfers varied. For example, participant 210 did not transfer at all in the retellings, while 205 did more frequently with primary mouth transfers from English.

Such CLI patterns found and described above for M1 learners were not found in M2 learners. As the M2 learners have no previous knowledge of any sign language, their CLI patterns are different, i.e., no sign language source. The CLI patterns observed for M2 learners were more linked to their L1 Swedish to various degrees and possibly their gestural repertoire. For example, Mesch and Schönström (2021) on the study of mouth actions using the same M2 data, i.e., from the STS L2 corpus, reported a higher usage of mouthing in M2 learners in terms of a higher frequency of full mouthing rather than using reduced mouthing (as in L1 signers).

## Discussion

The purpose of this study was to explore the role of multimodality in the acquisition of sign lexicon in two groups of learners, of which one, M1 learners, had prior knowledge of a sign language, and the other did not, i.e., are M2 learners. CLI has been used as a framework to examine patterns of CLI in the retold narratives produced by the groups. As a comparison, data from L1 signers were provided. The results revealed that the lexical distribution of M1 learners was more similar to that of L1 signers and more different from M2 learners, as M2 learners exhibit less use of depicting signs. This is in line with previous studies that have found spatial structures to be difficult to acquire for M2 learners (e.g., McKee and McKee, 1992; Ferrara and Nilsson, 2017; Shield and Meier, 2018; Boers-Visker, 2020; Gulamani et al., 2020). In contrast, M2 learners show higher usage of lexical signs and fingerspelling. Furthermore, we found instances of cross-linguistic influence in the M1 group consisting of L1/Ln signs and variation in handshape configurations in lexical and depicting signs.

With respect to the above CLI observations in M1 and M2 signers, we can conclude that CLI is possible regardless of the modality difference, but it seems that the learners’ modality experiences elicit different types of transfer. Even if the modalities are fundamentally different channels for perception and production, there are superficial and conceptual similarities. Same-modal language transfer allows for the direct physical transfer of the signs, i.e., as with the lexical transfer of L1/Ln signs into L2 STS, as well as the partly lexical transfer of mouthing. Different-modal transfers allow for more superficial and structural transfers that influence STS production. The perceived cross-linguistic similarities between Swedish words and lexical signs and the use of fingerspelling in STS obviously create motivation or possibility for the M2 learners to use lexical signs and fingerspelling, which contributes to the over-production of such signs.

On the contrary, the shared modality of sign languages contributes a more modality-based transfer in M1 signers through higher use of depiction in M1 signers compared with M2 signers. The M1 narratives from the short film clip “The Plank” consisted, to a large extent, of depicting signs that are contextually bounded and spontaneously created at the moment they are expressed. The M1 participants’ production of such depicting signs can be seen as a positive transfer from the sign modality, i.e., the participants know how depicting signs are used in narratives and used these to a higher degree than lexical signs. Such a strategy works very well in this type of narrative; lexical signs are not required when retelling the actions in the clip, and thus, the production can be perceived as STS and “sign language” in general. However, we also found that the improvisation and use of many different depicting signs also meant that the participants used a range of different lexical signs (from both STS and other sign languages) for the same referent, particularly regarding the “beer glass.”

Our findings link to previous CLI findings for spoken languages. First, the differences between M1 and M2 signers in the distribution of sign types could be linked to the assumption about the learners’ perceived similarity of the source and recipient language. However, the perception of the similarities is configured differently between M1 and M2 learners. As M1 learners can resort to modality experience of at least one sign language, it allows for a positive transfer of modality-specific structures, such as the use of depicting signs. At the same time, M1 learners underuse fingerspelling, as this kind of knowledge is probably associated with knowledge of Swedish, i.e., it requires a level of multilingual competence. M2 learners, in contrast, do not have any experience of the sign modality (except some possible gestural knowledge) but resort to the L1 knowledge of Swedish. Thus, their sign distribution demonstrates an overproduction of lexical signs and fingerspelling as a result of the perceived similarity between spoken Swedish words and lexical signs and fingerspelling.

Furthermore, transfers based on sign modality were observed in M1 learners through lexical transfers, mouthing transfers, and handshape transfers. Interestingly, the qualitative analysis shows that it could be linked to forward and lateral transfers. Knowledge of international sign and/or ASL (as lingua franca) among some M1 participants also contributed to the lateral transfer of a few signs and mouthing transfers. However, interferences based on a forward transfer were hard to find. However, we observed some handshape transfers that could be a type of forward transfer (e.g., the Russian handshape for “window”) and lexical transfer (e.g., transfer of Lithuanian sign of TREE).

Regarding the quantitative results, M1 learners use, as mentioned above, depicting signs to a greater extent than M2 learners and in a manner comparable to the baseline L1 signers (as the difference between M1 and L1 was not statistically significant). We believe this is an instance of CLI here as well. The M1 learners are still in the process of learning the lexical signs. In the meanwhile, the higher use of depicting signs may cover the M1 learners’ limited knowledge of lexical signs. Since they have access to previous knowledge of how to use depicting signs, this is positively transferred to the L2 STS. In contrast, M2 learners have limited previous knowledge of using depicting signs. Instead, they rely on the “one word–one sign” learning strategy as it has an observed similarity that STS and Swedish share. Thus, both M1 and M2 learners rely on perceived similarities of STS to their L1 but in different ways. However, it should be noted that we have not considered any form of accuracy analysis in this study. Instead, we have focused on the performance of the sign categories only through use and distribution.

An analysis of qualitative aspects of negative transfer in the M2 group was harder to conduct. For instance, in our initial analysis, we noticed using fingerspelling and the use of lexical signs that are prepositions. Nevertheless, it was not entirely straightforward to mark them as non-target forms, i.e., as negative transfers, as such usage of fingerspelling and prepositions is apparent in the L1 group and part of the language contact between STS and STS Swedish. Instead, as mentioned, we have departed from the frequency and distribution to illustrate the M2 groups’ usage of sign categories from the lens of CLI. However, future studies focusing on syntactic production may reveal interesting results regarding the use of prepositions in the M2 group, for example. The negative transfers in M1 were more apparent as they were, in fact, non-target forms.

In our study, the number of gestures was low among all three groups. The M2 group used the largest amount, but these only consisted of 3.0% of the total number of signs. L1 used the lowest number of gestures, only 1.6%, and the M1 group 2.6%. However, although not statistically significant, the slight difference between M2 and L1 signers *may* indicate that M2 signers transfer some of their gestural knowledge when producing narratives. Nor could any difference be found among the three groups in the sign category pointings. These results may be somewhat surprising for gestures and pointings because they are common strategies in both sign languages and as co-speech gestures. It may be caused by the movie clip “The Plank” being only 1 min long, and the content does not elicit gestures and pointings but rather lexical signs and depicting signs. If the groups had produced a longer narrative or their own stories, the gestures and pointings might have been more frequent. Future studies may reveal if this is the case and, if so, if there are group differences.

This study has focused on a particular group of M1 learners to be able to compare with M2 learners as equally as possible. Consequently, we focused on M1 learners with a comparable L1 background and educational background as the M2 learners. Still, there is a good amount of variability within the M1 group in terms of their L1 SLs and age. Furthermore, it was challenging to recruit enough participants to provide a good picture of the M1 acquisition. In our project Mulder, studying deaf migrants, there are a considerable number of participants not included here, with diverse linguistic and educational backgrounds that would not fit within the frame of this study.

It should be worth highlighting some individual (and group) variations associated with our M1 and M2 participants. First, regarding the learning time of L2 STS, it could be noted that some of the M1 learners have a shorter time of their learning of STS compared with the M2 learners. In addition, M1 learners are learning (written) Swedish simultaneously, while M2 learners are native speakers of Swedish. This fact can explain why the usage of fingerspelling is lower in the M1 group compared to the M2 (and L1) group. This also supports that lexical transfers of other sign languages are apparent in the M1 group and that the M2 group has an overproduction of mouthing, as previously reported (Mesch and Schönström, 2021). However, exposure time to STS (and other sign languages) may be much larger for the M1 participants compared with the M2 participants. Second, education level and social status may matter. All M2 learners are, in fact, students at the university level and hearing, i.e., they have a more privileged position in the Swedish society compared with the M1 learners. But it is not clear how this would affect their signing. Most M1 students also have some kind of education after the elementary level but no university level education. However, M1 learners benefit from cultural-bound access to the deaf community, as most participants seem to be building connections to the Swedish Deaf community. It is a larger step for a hearing M2 learner to get involved in this community, if possible. Furthermore, as earlier SLA research indicates, motivation matters and cultural-related motivation boosts learning a new language. Third, the cultural boundness and knowledge about international meetings between deaf people can also influence the M1 learners’ signing. It can be found in terms of their higher use of depicting signs and the qualitative lexical and mouthing transfers we have accounted for in the result section, even if unconscious, at least with regard to handshape transfer. Finally, even though it is exceptionally hard to collect enough data from a group of sign language learners with a fully comparable background, especially for the M1 group, it is important to consider these individual and group variations in our results. This is also something future studies should consider.

To conclude, this study has shown that M2 learners exhibit cross-modal cross-linguistic transfer from Swedish (through higher usage of lexical signs and fingerspelling) and that M1 learners exhibit same-modal cross-linguistic transfer from L1/Ln sign languages through higher usage of depicting signs and use of signs from L1/Ln sign language and international signs. Furthermore, the study suggests that the modality-specific lexicon is harder for M2 learners to acquire despite possible underlying gestural knowledge. In contrast, M1 learners have access to modality-specific knowledge, overlapping access to gestural knowledge, and iconicity, which facilitates the modality-specific use of the lexicon and is open for direct lexical transfer from other sign languages. Thus, second language learning seems to be based on multimodality and multimodal competence, as well as multilingual competence. However, this study has only focused on the production of the lexicon. For future studies, it would be interesting to broaden the scope of possible CLI on other structures, especially from a morphosyntactic perspective, to see whether different grammatical profiles of spoken and sign languages influence the learning of the languages.

## Data Availability Statement

The raw data supporting the conclusions of this article will be made available by the authors, without undue reservation.

## Ethics Statement

The studies involving human participants were reviewed and approved by the Swedish Ethical Review Authority (DNR 2020-02865 and 2013/5:8). The patients/participants provided their written informed consent to participate in this study. Written informed consent was obtained from the individual(s) for the publication of any identifiable images or data included in this article.

## Author Contributions

KS designed the study and responsible for the analysis work. KS and IH collected the data and involved in manuscript drafting and revisions. Both authors contributed to the article and approved the submitted version.

## Conflict of Interest

The authors declare that the research was conducted in the absence of any commercial or financial relationships that could be construed as a potential conflict of interest.

## Publisher’s Note

All claims expressed in this article are solely those of the authors and do not necessarily represent those of their affiliated organizations, or those of the publisher, the editors and the reviewers. Any product that may be evaluated in this article, or claim that may be made by its manufacturer, is not guaranteed or endorsed by the publisher.
